# Impact of Surface Modification with Gold Nanoparticles on the Bioelectrocatalytic Parameters of Immobilized Bilirubin Oxidase

**Published:** 2014

**Authors:** D. V. Pankratov, Y. S. Zeifman, A. V. Dudareva, G. K. Pankratova, M. E. Khlupova, Y. M. Parunova, D. N. Zajtsev, N. F. Bashirova, V. O. Popov, S. V. Shleev

**Affiliations:** A.N. Bach Institute of Biochemistry of Russian Academy of Sciences, Leninsky Ave. 33, building 2, 119071 Moscow, Russia; National Research Center “Kurchatov Institute”, Akademika Kurchatova Sq. 1, 123182 Moscow, Russia; I.G. Petrovsky Bryansk State University, Bezhitskaya St. 14, 241036 Bryansk, Russia

**Keywords:** gold nanoparticle, bilirubin oxidase, direct electron transfer, bioelectrocatalysis

## Abstract

We unveil experimental evidence that put into question the widely held notion concerning the impact of nanoparticles on the bioelectrocatalytic parameters of enzymatic electrodes. Comparative studies of the bioelectrocatalytic properties of fungal bilirubin oxidase from *Myrothecium verrucaria *adsorbed on gold electrodes, modified with gold nanoparticles of different diameters, clearly indicate that neither the direct electron transfer rate (standard heterogeneous electron transfer rate constants were calculated to be 31±9 s^-1^) nor the biocatalytic activity of the adsorbed enzyme (bioelectrocatalytic constants were calculated to be 34±11 s^-1^) depends on the size of the nanoparticles, which had diameters close to or larger than those of the enzyme molecules.

## INTRODUCTION

Numerous studies have reported on effective direct electron transfer (DET ) of various enzymes (including blue multicopper oxidase – MCO) immobilized on the surface of nanostructured electrodes with metal and carbon nanoparticles, carbon nanotubes, graphene, etc. [1-3]. An increase in the bioelectrocatalytic current when using nanostructured surfaces was regarded as the defining evidence behind the acceleration of the DET reaction in these studies; however, neither a quantitative comparative analysis of DET based on a voltammograms analysis, nor a calculation of the standard constants of the heterogeneous electron transfer reaction (*k*0) has been performed. Moreover, there are serious discrepancies in the data even for a single enzyme (in particular, laccase from the *Trametes hirsuta* (*Th*Lc) fungus immobilized on the gold surface). For instance, the use of AuNP and nanoporous gold helped to increase DET [4], whereas an extremely low bioelectrocatalytic activity of the enzyme was observed for DET [5] in nano/microstructured silicon chips modified with gold with the enzyme immobilized on their surface. Since the use of bioelectrodes without a nano-modified surface leads to a very low heterogeneous transfer rate and sometimes to a complete absence of DET , the routine explanation for enzyme “nanobinding” is the orientation of the enzyme on the nanostructured surface, which contributes to DET .

Despite the fact that a possible dependence of *k*_0_ on the size of the metal or carbon nanoparticles has yet to be studied and that the two opposite dependences of the bioelectrocatalytic oxygen reduction current on the diameter of AuNP on electrodes modified with MCO were recently demonstrated (*e.g.*, [6]), the belief remains that the size of AuNP used for electrode nanomodification is a very important factor that determines ET in the reactions between a redox enzyme and the electrode surface. This study offers experimental results that demonstrate the unlikeliness of this hypothesis.

## EXPERIMENTAL

**Materials and Methods**

Na_2_HPO_4_∙2H_2_O, NaH_2_PO_4_·H2_O_, NaCl, HAuCl_4_·3H_2_O, H_2_O_2_, H_2_SO_4_, NaBH_4_, and sodium citrate were purchased from Sigma-Aldrich GmbH (Germany) and used without further purification. Oxygen was acquired from AGA Gas AB (Sweden). Buffers and other solutions were prepared using deionized water (18 MΩ∙cm) produced using a PURE LAB UHQ II system (ELGA Labwater, UK). All experiments were performed at room temperature in PBS (pH 7.4) consisting of a 50 mM HPO_4_^2-^ /H_2_PO_4_ - solution containing 150 mM NaCl.

*Mv*BOx was a gift from Amano Enzyme Inc. (Japan).

Electrochemical measurements were performed using a μAutolab Type III/FRA2 potentiostat/galvanostat (Metrohm Autolab BV, the Netherlands) using a three-electrode circuit with a saturated calomel reference electrode (242 mV *vs. *normal hydrogen electrode, NHE) and a platinum wire as an auxiliary electrode.

Sonication was performed using a Ultrasonic Cleaner XB2 bath (VWR International Ltd., UK). SEM was performed on a FEI Nova NanoLab 600 high-resolution scanning electron microscope (the Netherlands). Spectrophotometric studies were carried out using a PharmaSpec UV-1700 UV-visible spectrophotometer (China).

Nanoparticles with a diameter of 5 to 60 nm were synthesized to study the impact of the AuNP size on the biocatalytic properties of *Mv*BOx.

**Synthesis of gold nanoparticles with a preset particle size**

**Fig. 1:**
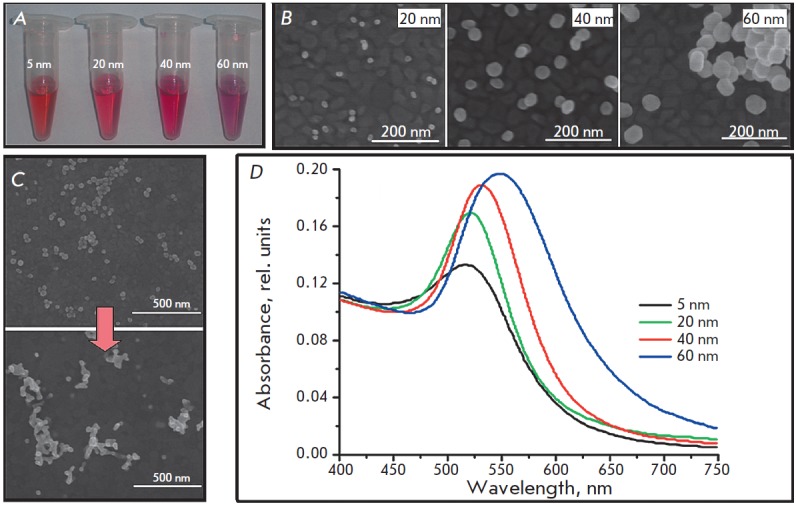
A. Photos of the colloidal solutions of synthesized AuNPs; B. SEM images of AuNP/Au samples; C. SEM image of AuNP_40_/Au before (top) and after (bottom) 2 cycles in H_2_SO_4_: D. Absorbance spectra of AuNP suspensions with different diameters

AuNP with the expected diameter of 5 nm (AuNP_5_, *Fig. 1A*) were synthesized as described in [7]. 50 ml of a 250 µM HAuCl_4_ solution was stirred for 1 min at room temperature, then 1111 µl of a 38.8 mM sodium citrate solution was added to the initial solution, and the mixture was stirred for another 1 min. Next, 555 µl of a freshly prepared 0.075% (wt.) NaBH_4_ solution in a 38.8 mM sodium citrate solution was poured into the reaction mixture and the solution was stirred for another 5 min. ;

AuNP with a diameter of 20–60 nm (*Fig. 1A*) were synthesized using sodium citrate as a reductant. 50 ml of a 250 µM HAuCl_4_ solution was brought to boil under constant stirring; 750, 500, or 260 µl of a 1% (wt.) sodium citrate solution was subsequently added to obtain AuNP with diameters of 20, 40, and 60 nm (AuNP_20_, AuNP_40_ and AuNP_60_), respectively. After adding sodium citrate, the mixture was incubated for 10 min under constant stirring without heating.

The diameter of the resulting nanoparticles was evaluated spectrophotometrically in accordance with the procedure described in [7] using the wavelength of maximum absorbance (*A*_spr_, *Fig. 1D*). The diameter of a AuNP less than 35 nm in size was calculated using the *A*_spr_/*A*_450_ ratio.

Nanoparticles 20-60 nm in size were further identified using SEM. The samples for microscopy were prepared by applying a small amount of AuNP obtained from diluted colloidal solutions over the flat surface of gold electrodes (*Fig. 1B*). It should be emphasized that the surface structure of the electrodes used in further studies was fundamentally different from that shown in *Fig. 1* due to a significantly higher amount of applied AuNP and surface changes resulting from treatment with H_2_SO_4_ (see below); thus, it can only be used to evaluate the diameters of the synthesized AuNP.

The results of a size evaluation of the nanoparticles produced through independent methods are shown in Table 1.

**Table 1:** Comparative analysis of the diameters of synthesized AuNP as determined using different methods

Diameter of AuNP, nm		
Expected	Determined spectrophotometrically	Determined using the SEM data
20	16	19±2
40	42-51	38±5
60	77	59±5

The data shown in *Figs. 1B,D *allow one to conclude that a direct determination of the AuNP size using the SEM method provides the most accurate and consistent data suitable for a statistical evaluation of the particle size distribution. However, this method has sensitivity limitations; in particular, in this case it was impossible to estimate the size of nanoparticles smaller than 10 nm. ;

All the prepared AuNP solutions, except for AuNP with a diameter of 5 nm, were concentrated by centrifugation at 10,000 g for 30 min. 95% of the supernatant was removed, and the AuNP precipitate was re-suspended using sonication. Nanoparticles 5 nm in diameter could not be concentrated using the proposed method; thus, the diluted solution was used for further experiments.

**Purification of gold electrodes and their modification with AuNP**

Polycrystalline gold disc electrodes (Bioanalytical Systems, USA) with a geometric surface area of 0.031 cm^2^ were mechanically cleaned through polishing with Microcloth paper (Buehler, UK) in an aluminum oxide suspension with a particle size of 0.1 µm (Struers, Denmark) to obtain a mirror surface. The electrodes were further washed with deionized water and electrochemically purified through cycling in 0.5 M H_2_SO_4_ using a range of potentials from –0.1 to +1.9 V *vs. *NHE for 20 cycles at a scan rate of 0.1 V∙s_-1_, then they were washed with water and dried in an air stream.

Following this, 5 µl of the solution (for concentrated suspensions) or 6 µl of the solution (for an AuNP suspension with a particle diameter of 5 nm (AuNP_5_)) was applied to the cleaned gold electrode surface. The modified electrode was then dried at room temperature. The AuNP modification procedure was repeated twice for concentrated suspensions of nanoparticles and 5 times for AuNP_5_. The obtained electrodes were cycled in 0.5 M H_2_SO_4_ with the potential ranging from 0.0 to +1.9 V *vs. *NHE. Two cycles were performed in order to avoid desorption and/or agglomeration of nanoparticles on the surface (*Fig. 1C*) at a scan rate of 0.1 V∙s^-1^. The electrodes were then washed with water and dried. The electrochemically active (real) surface area of the electrodes (*A*_real_) was calculated according to [8], assuming the level of the charge required for the reduction of gold oxide during electrochemical cycling under specified conditions to be equal to 390 ± 10 µC ∙cm^-2^ [9].

The results of the calculation of *A*real presented in Table 2 show no direct relationship between *A*real and the size of the AuNP used for surface modification. This fact indirectly confirms earlier results [10] on the formation of a disordered three-dimensional structure via repeated cycling of the AuNP/Au electrode in 0.5 M H_2_SO_4_. In connection to this, two types of AuNP/Au electrodes were used in further experiments: either treated with H_2_SO_4_ (m-AuNP/Au) or without cycling (u-AuNP/Au). *A*_real_ for u-AuNP/Au electrodes was assumed to be equal to *A*_real_ for m-AuNP/Au samples.

**Table 2:** Real surface area vs. nanoparticles size

AuNP diameter, nm	Real surface area, cm^-2^
5	0.21±0.01
20	1.40±0.01
40	1.25±0.05
60	1.23±0.03

Biomodification of the surface of AuNP/Au electrodes was carried out through direct adsorption of the enzyme for 20 min from a *Mv*BOx solution with a protein concentration of 0.25 mg∙ml^-1^. The surface concentration of the enzyme was assumed to be 3.0 pmol∙cm^-2^.

The *k*_0_ and *k_cat_^app^* values were calculated using the MathCAD 14 software package and the equation:

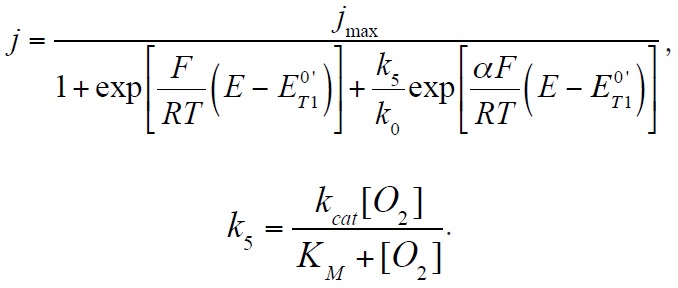



The kinetic scheme of enzyme functioning used to establish the equation was presented in [11].

## RESULTS AND DISCUSSION

**Fig. 2:**
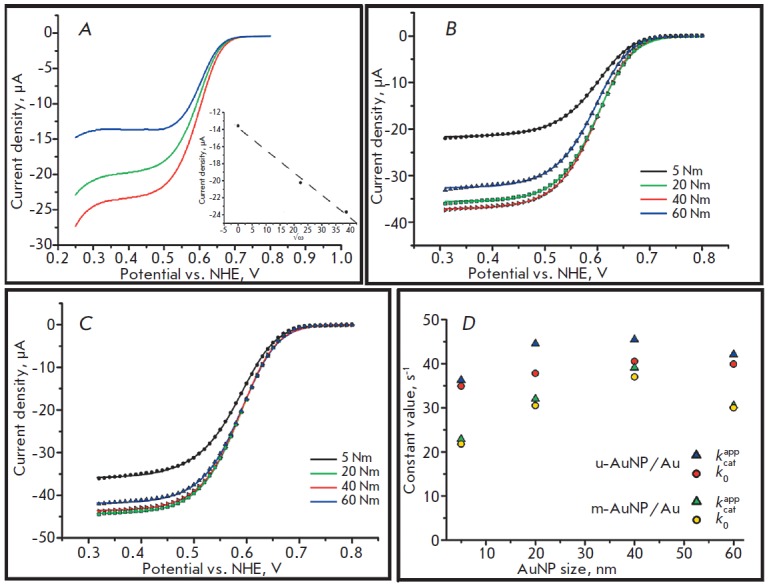
A) Cyclic voltammograms (cathodic waves) of biomodified m-AuNP_20_/Au electrodes recorded at different rotation rates, rpm: 0 (blue), 500 (green) and 1500 (red). Inset – current density at 0.35 V as a function of ω^1/2^; B), C). Cyclic voltammograms (cathodic waves) of *Mv*BOx modified m-AuNP/Au (B) and u-AuNP/Au (C) electrodes based on AuNPs of different diameters; D) Dependences of the calculated bioelectrocatalytic parameters on the size of AuNPs. Conditions for all CVs: oxygen saturated PBS, scan rate – 20 mV s^-1^, second cycle

The bioelectrodes were placed in oxygenated PBS, followed by CV recording at an electrode rotation speed of 1,500 min^-1^ to eliminate possible diffusion limitations (*Fig. 2A*). A pronounced bioelectrocatalytic response with an initial oxygen electrical reduction potential of about 0.75 V was registered for all the electrodes used. As illustrated in *Fig. 2 B,C*, substantially similar *j*_max_ values were obtained using the electrodes biomodified with m-AuNP/Au (31.4 ± 5.9 µA∙cm^-2^ ) and u-AuNP/ Au (43.4 ± 5.6 µA∙cm^-2^) electrodes.

Taking these data into account, the *k*_0_ (31 ± 9 s^-1^) and* k_cat_^app^*(34 ± 11 s^-1^) values were calculated. The results are shown in *Fig. 2D*. The calculated *k*_0_ and *k_cat_^app^* values are similar regardless of the AuNP diameter and the electrode type used. The similarity of the constants for the electrodes based on m-AuNP/Au and u-AuNP/Au indicates that the assumption of identity of the *A*_real_ (despite different structures) for both types of samples does not add a critical error to the calculations. The overestimated constants for u-AuNP/Au attest to the slightly higher surface area of those electrodes compared to m- AuNP/Au, owing to the absence of AuNP agglomerates (*Fig. 1C*), which also reduces the *A*_real_. The pronounced bends of constant *vs. *AuNP size profiles observed when using m-AuNP/Au may be due to the surface modification because of the formation of different three-dimensional (3D) structures during the treatment of electrodes in H_2_SO_4_. The behavior of the enzyme on these heterogeneous surfaces cannot be fully described by the single theory used in this study to calculate biocatalytic parameters without introducing additional corrections. Moreover, the formation of 3D agglomerates yields errors when a single value of the surface concentration of the enzyme (3.0 pmol∙cm^-2^) is used for all the m-AuNP/ Au-based bioelectrodes. This fact can also explain the shape of the curves. However, based on the experimental data and simulation results, we can affirm that the bioelectrocatalytic properties of *Mv*BOx immobilized on the AuNP/Au surface show no dependence on the nanoparticle diameter. The increased electrocatalytic current of the bioelectrodes modified with nanoparticles of different sizes found in a number of previous studies is most likely associated with an increase in the geometrical surface area rather than the acceleration of the DET reactions or an increase in the bioelectrocatalytic constants of the immobilized enzymes.

## CONCLUSIONS

Our results have experimentally demonstrated no relationship between the bioelectrocatalytic parameters of *Mv*BOx immobilized on the AuNP/Au surface and the nanoparticle diameter. However, it should be noted that the results obtained in this study cannot be extrapolated to other nanobiomodified surfaces (*e.g.*, other nanoparticles and redox enzymes). In particular, it is of particular interest to study the impact of nanoparticles with a diameter lower than the size of the enzyme that can promote electron transfer between the enzyme and the electrode surface. Such experiments will provide a more complete picture of the impact of nanoparticles on the bioelectrocatalytic parameters of oxidoreductases.

## Abbreviations

3D: three-dimensional
A(real): real/electrochemical surface area
CV: cyclic voltammogram
ET: electron transfer
DET: direct electron transfer
k(0): standard heterogeneous electron transfer rate constant
k(app/cat): apparent bioelectrocatalytic constant
MCO: blue multicopper oxidase
MvBOx: Myrothecium verrucaria bilirubin oxidase
AuNP(n): gold nanoparticles with diameter of n nm
AuNP(n)/Au: gold electrode modified with AuNP(n) before and after cycling in sulfuric acid (m-AuNP/Au u-AuNP/Au, respectively)
ThLc: Trametes hirsuta laccase
PBS: phosphate buffered saline
NHE: normal hydrogen electrode
SEM: scanning electron microscopy
A(spr): absorbance maximum
A(450): absorbance at the wavelength of 450 nm
Γ: enzyme surface concentration
j(max): maximum current density
